# European traditional tomatoes galore: a result of farmers’ selection of a few diversity-rich loci

**DOI:** 10.1093/jxb/erac072

**Published:** 2022-03-31

**Authors:** Jose Blanca, Clara Pons, Javier Montero-Pau, David Sanchez-Matarredona, Peio Ziarsolo, Lilian Fontanet, Josef Fisher, Mariola Plazas, Joan Casals, Jose Luis Rambla, Alessandro Riccini, Samuela Palombieri, Alessandra Ruggiero, Maria Sulli, Stephania Grillo, Angelos Kanellis, Giovanni Giuliano, Richard Finkers, Maria Cammareri, Silvana Grandillo, Andrea Mazzucato, Mathilde Causse, Maria José Díez, Jaime Prohens, Dani Zamir, Joaquin Cañizares, Antonio Jose Monforte, Antonio Granell

**Affiliations:** Instituto de Conservación y Mejora de la Agrodiversidad Valenciana (COMAV-UPV), Universitat Politècnica de València, València, Spain; Instituto de Conservación y Mejora de la Agrodiversidad Valenciana (COMAV-UPV), Universitat Politècnica de València, València, Spain; Instituto de Biología Molecular y Celular de Plantas (IBMCP), Consejo Superior de Investigaciones Científicas (CSIC), Universitat Politècnica de València, València, Spain; Instituto de Conservación y Mejora de la Agrodiversidad Valenciana (COMAV-UPV), Universitat Politècnica de València, València, Spain; Instituto de Conservación y Mejora de la Agrodiversidad Valenciana (COMAV-UPV), Universitat Politècnica de València, València, Spain; Instituto de Conservación y Mejora de la Agrodiversidad Valenciana (COMAV-UPV), Universitat Politècnica de València, València, Spain; INRAE, UR1052, Génétique et Amélioration des Fruits et Légumes, Centre de Recherche PACA, Domaine Saint Maurice, 67 Allée des Chênes, CS 60094, 84143 Montfavet, France; Robert H. Smith Institute of Plant Sciences and Genetics in Agriculture, The Hebrew University of Jerusalem, Rehovot, Israel; Instituto de Conservación y Mejora de la Agrodiversidad Valenciana (COMAV-UPV), Universitat Politècnica de València, València, Spain; Department of Agri-Food Engineering and Biotechnology/Miquel Agustí Foundation, UPC-BarcelonaTech, Campus Baix Llobregat, Esteve Terrades 8, 08860 Castelldefels, Spain; Instituto de Biología Molecular y Celular de Plantas (IBMCP), Consejo Superior de Investigaciones Científicas (CSIC), Universitat Politècnica de València, València, Spain; Department of Agriculture and Forest Sciences (DAFNE), Università degli Studi della Tuscia, Viterbo, Italy; Institute of Biosciences and BioResources (IBBR), National Research Council of Italy (CNR), Via Università 133, 80055 Portici, Italy; Institute of Biosciences and BioResources (IBBR), National Research Council of Italy (CNR), Via Università 133, 80055 Portici, Italy; Italian National Agency for New Technologies, Energy and Sustainable Economic Development (ENEA), Casaccia Research Centre, Rome, Italy; Institute of Biosciences and BioResources (IBBR), National Research Council of Italy (CNR), Via Università 133, 80055 Portici, Italy; Group of Biotechnology of Pharmaceutical Plants, Laboratory of Pharmacognosy, Department of Pharmaceutical Sciences, Aristotle University of Thessaloniki, 54124 Thessaloniki, Greece; Italian National Agency for New Technologies, Energy and Sustainable Economic Development (ENEA), Casaccia Research Centre, Rome, Italy; Plant Breeding, Wageningen University and Research, POB 386, 6700 AJ Wageningen, The Netherlands; Institute of Biosciences and BioResources (IBBR), National Research Council of Italy (CNR), Via Università 133, 80055 Portici, Italy; Institute of Biosciences and BioResources (IBBR), National Research Council of Italy (CNR), Via Università 133, 80055 Portici, Italy; Department of Agriculture and Forest Sciences (DAFNE), Università degli Studi della Tuscia, Viterbo, Italy; INRAE, UR1052, Génétique et Amélioration des Fruits et Légumes, Centre de Recherche PACA, Domaine Saint Maurice, 67 Allée des Chênes, CS 60094, 84143 Montfavet, France; Instituto de Conservación y Mejora de la Agrodiversidad Valenciana (COMAV-UPV), Universitat Politècnica de València, València, Spain; Instituto de Conservación y Mejora de la Agrodiversidad Valenciana (COMAV-UPV), Universitat Politècnica de València, València, Spain; Robert H. Smith Institute of Plant Sciences and Genetics in Agriculture, The Hebrew University of Jerusalem, Rehovot, Israel; Instituto de Conservación y Mejora de la Agrodiversidad Valenciana (COMAV-UPV), Universitat Politècnica de València, València, Spain; Instituto de Biología Molecular y Celular de Plantas (IBMCP), Consejo Superior de Investigaciones Científicas (CSIC), Universitat Politècnica de València, València, Spain; Instituto de Biología Molecular y Celular de Plantas (IBMCP), Consejo Superior de Investigaciones Científicas (CSIC), Universitat Politècnica de València, València, Spain; CONICET-National University of La Plata, Argentina

**Keywords:** Crop evolution, diversification, fruit morphology, genome-wide association study, genotyping by sequencing, selection, single nucleotide polymorphism

## Abstract

A comprehensive collection of 1254 tomato accessions, corresponding to European traditional and modern varieties, early domesticated varieties, and wild relatives, was analyzed by genotyping by sequencing. A continuous genetic gradient between the traditional and modern varieties was observed. European traditional tomatoes displayed very low genetic diversity, with only 298 polymorphic loci (95% threshold) out of 64 943 total variants. European traditional tomatoes could be classified into several genetic groups. Two main clusters consisting of Spanish and Italian accessions showed higher genetic diversity than the remaining varieties, suggesting that these regions might be independent secondary centers of diversity with a different history. Other varieties seem to be the result of a more recent complex pattern of migrations and hybridizations among the European regions. Several polymorphic loci were associated in a genome-wide association study with fruit morphological traits in the European traditional collection. The corresponding alleles were found to contribute to the distinctive phenotypic characteristic of the genetic varietal groups. The few highly polymorphic loci associated with morphological traits in an otherwise a low-diversity population suggests a history of balancing selection, in which tomato farmers likely maintained the morphological variation by inadvertently applying a high selective pressure within different varietal types.

## Teaser

The high phenotypic diversity observed among European traditional tomato varieties was created by traditional farmer-driven selection by inadvertently combining a very few polymorphic loci subjected to balancing selection.

## Introduction

The widespread crop plant tomato (*Solanum lycopersicum* L. var. *lycopersicum*; SLL) originated in Mesoamerica, in a region corresponding to present-day Mexico, as a result of domestication of the ancestor *S. lycopersicum* L. var. *cerasiforme* (SLC) (Blanca *et al*., 2012, 2015; Razifard *et al.*, 2020). Tomato was later imported to Europe. The Italian botanist Mattioli described varieties with flat, round, and segmented fruit types in 1544 (McCue, 1952). This indicated that tomato fruits of various shapes had probably arrived to Europe from America (Sahagún, 1577; Luckwill, 1943; Sanfunentes Echevarrría, 2006). The tomato was not immediately adopted for consumption by Europeans as it was considered at different times and regions as poisonous, an aphrodisiac, ornamental, valuable for sauces and soups, a miracle cure, and, finally, a fresh salad ingredient (Harvey *et al.*, 2003). It was only as late as the mid-19th century that the tomato became a regular component of the diet in Britain and North America (Harvey *et al.*, 2003). By contrast, the tomato was better received, extensively cultivated, and consumed as food by the 18th century in Southern Europe, which therefore could have become a secondary center of diversity (Boswell, 1937; Bauchet and Causse, 2012). As a result of this long tradition of use, a large number of traditional varieties of tomato are currently available along the Mediterranean basin, showing an impressive phenotypic diversity in terms of fruit appearance, adaptation to local conditions, and culinary use. Despite the interest in unveiling the population history and the processes that gave rise to the domestication of tomato (Blanca *et al.*, 2015; Razifard *et al.*, 2020), there are as yet no detailed genetic analyses of the diversification history of the European traditional tomato varieties.

The extent and type of the molecular variation in the tomato clade has been extensively analyzed in previous studies. The first molecular studies, carried out with isoenzymes, determined that the worldwide cultivated SLL was less variable than the wild *Solanum pimpinellifolium* (SP) and that the ancestral SLC (which includes wild, feral, and semi-domesticated accessions) was genetically closer to SLL than to SP (Rick *et al.*, 1974; Rick and Fobes, 1975). A clear trend of diversity reduction was observed at the species/subspecies level, probably due to bottlenecks associated with migrations and to the selection pressure imposed by humans during the early stages of domestication and the development of cultivars from SP to SLC, and, lastly, to SLL (Blanca *et al*., 2012, 2015; Razifard *et al.*, 2020).

Several molecular studies have unveiled the worldwide genetic structure within SLL and divided it into four major groups: processing, fresh market, cherry, and traditional tomatoes (Williams and Clair, 1993; Robbins *et al.*, 2011; Sim *et al.*, 2011; Casals *et al.*, 2019). The first three groups corresponded to modern tomato varieties created by breeders in the 20th century, characterized by their different culinary uses and by the introgression of genes from wild species, mainly to increase disease resistance and also to develop new cultivars. In contrast, traditional cultivars (in this study, vintage, landraces, and heirlooms are considered synonymous with traditional) are defined as those that were developed by traditional farmers by intuitive breeding and that were cultivated (indeed, some of them are still cultivated locally nowadays) before the advent of systematic breeding programs. Park *et al.*, (2004) found genetic differentiation between traditional and modern cultivars. More comprehensive analyses using the Solanaceae Coordinated Agricultural Project (SolCAP) genotyping panel, consisting of 7720 single nucleotide polymorphisms (SNPs) (Sim *et al.*, 2012a), confirmed the previously described fresh, processing, and traditional groups (Blanca *et al*., 2012, 2015; Sim *et al.*, 2012a). These studies also found two extra clusters, located between SLL and SP, corresponding to cultivated and wild cherry tomatoes (Sim *et al.*, 2012a), and clarified the status of the cherry tomatoes (Blanca *et al*., 2012, 2015)—some of them being SLC originating from South America, Mesoamerica, and the subtropical regions, and the others being modern cherry tomatoes obtained by hybridizing cultivated SLL with wild SP. In addition, rarefaction analysis revealed that traditional SLL and SLC from outside Peru and Ecuador had much lower genetic diversity than Peruvian and Ecuadorian SP and SLC (Blanca *et al.*, 2015).

The above-mentioned studies differentiated the modern varieties from the traditional ones, but none of them found any structure within the traditional tomato group. A few studies have addressed the differentiation of traditional variety groups, mostly among Spanish (García-Martínez *et al.*, 2006) and Italian collections (Mazzucato *et al.*, 2008; Sacco *et al.*, 2015; Esposito *et al.*, 2020). These studies have focused on a limited number of accessions from a narrow local diversity, and therefore, a broader view is clearly needed to better understand the history and relationships of the European traditional varieties.

In the present study, the genomes of 1254 European tomato accessions collected from Southern European seed banks were partially sequenced by genotyping by sequencing (GBS) (Baird *et al.*, 2008; Elshire *et al.*, 2011). The genetic structure, diversity, and association between the polymorphic loci and the morphological variations in these accessions were analyzed to shed light on the history of the make-up of the diverse traditional European tomatoes.

## Materials and methods

### Materials

A total of 1254 tomato accessions were analyzed in this study. Of these accessions, 1044 are part of the collection of the EU TRADITOM project (www.traditom.eu). Seeds comprising the TRADITOM collection were obtained from the gene banks of the Institute for the Conservation and Improvement of Valencian Agrodiversity at the Polytechnic University of Valencia (COMAV-UPV, Valencia, Spain), the Balearic Island University (UIB, Mallorca, Spain), the Station d’Amelioration des Plantes Maraicheres of the French National Institute for Agricultural Research, (INRA, Montfavet, France), the Department of Agriculture and Forest Sciences of the University of Tuscia (UNITUS, Viterbo, Italy), the Institute of Biosciences and Bioresources of the Italian National Council of Research (CNR-IBBR, Portici, Italy), the Agricultural Research Center of Macedonia and Thrace of the National Agricultural Research Foundation (GGB-NAGREF, Thessaloniki, Greece), and the seed collections of the Miquel Agustí Foundation of the Polytechnic University of Catalunya (FMA-UPC, Castelldefels, Spain), BioEconomy of the Italian National Council of Research (CNR-IBE, Catania, Italy), ARCA 2010 a.r.l Soc.Coop. (ARCA, Acerra, Italy), the University of Reggio Calabria (UNIRC, Reggio Calabria, Italy), and the Robert H. Smith Faculty of Agriculture, Food and Environment of The Hebrew University of Jerusalem (HUJI-ARO, Rehovot, Israel). An additional set of 110 accessions, obtained from the COMAV gene bank (COMAV-UPV, Valencia, Spain), was used as wild and semi-domesticated accessions. This set contained 10 wild accessions from the Galapagos Islands, one accession of each wild species *Solanum habrochaites*, *Solanum chmielewskii*, and *Solanum peruvianum*, 39 *S. pimpinellifolium* accessions from Peru (SP) and North Ecuador (SP_NECu), and 34 SLC accessions, 19 traditional accessions, four modern accessions, and one *S. pimpinellifolium* × *S. lycopersicum* hybrid (SP×SL), corresponding to cherry cultivars and other crosses between the two species. Passport data can be found in Supplementary Table S1. The germplasm collection was extensively phenotyped in the TRADITOM project (Pons *et al.*, 2017). The dataset corresponding to fruit morphology and color traits obtained in a trial at HUJI-ARO was used.

### DNA extraction, library preparation, and sequencing

Genomic DNA was isolated from young leaves of 5–10 seedlings per accession, using the DNeasy 96 Plant Mini Kit (Qiagen, Germany). Genotyping by sequencing (GBS) was performed following the procedure reported by Elshire *et al.*, (2011). Briefly, DNA was digested with the restriction enzyme *Ape*K I, barcoded libraries were prepared to track each accession, and the DNA sequence corresponding to the region flanking the *Ape*K I site was obtained on an Illumina HiSeq 2000 platform by LGC Genomics GmbH (Berlin, Germany). Following the Variant Call Format standard, we use the term ‘sample’ to refer to one genotyping experiment from one accession. The sequence data can be found in NCBI (https://www.ncbi.nlm.nih.gov/sra) under accession number PRJNA722111.

### Read mapping, SNP calling, and SNP filtering

FastQC was used to evaluate the quality of the sequenced reads. High-quality reads were mapped against the *S. lycopersicum* genome build 2.50 (Sato *et al.*, 2012) using BWA-MEM (Li, 2013, Preprint). After mapping, in order to avoid possible false positives caused by misalignment, the PHRED quality of three aligned nucleotides from each read end was set to 0 (Li, 2011). Mapping statistics were calculated with the samtools stats command (Li *et al.*, 2009).

SNP calling was carried out by freebayes (Garrison and Marth, 2012, Preprint) using the following parameters: a minimum mapping quality of 57, 5 best alleles, 20 minimum base quality, 0.05 maximum mismatch read alignment rate, 10 minimum coverage, 2 minimum alternate allele count, and 0.2 minimum alternate fraction. To avoid regions with potential assembly problems in the reference genome, the Heinz 1706 reads used to build the reference genome were mapped against the reference assembly version SL2.50. A 50X mean coverage was obtained. Any region having a coverage higher than 200X was removed from the SNP calling.

SNP and genotype processing were carried out by using the Python variation library deposited in the Zenodo repository (https://doi.org/10.5281/zenodo.5783972). All scripts developed for the analyses have been also uploaded to Zenodo (https://doi.org/10.5281/zenodo.5785413). To create the tier1 SNP set to be used in the rest of the analyses, the genotypes with a quality lower than 5 were set to missing. The variants with SNP quality lower than 50, an observed heterozygosity higher than 0.1, and a call rate lower than 0.6 were filtered out. Moreover, in order to avoid false positives, only variants in which the minor allele was found in more than two samples were kept. This filtering was carried out with the ‘create_tier1.py’ script. For some analyses, the pericentromeric regions, which seldom recombine, were removed. To locate the pericentromeric regions, a piecewise regression analysis was applied to the relationship between the genetic distance and the physical positions of the markers of the EXPIM map (Sim *et al.*, 2012b). Regression analyses were done using the segmented R library (Muggeo, 2003). The calculated pericentromeric regions were: chromosome 1, from 5488553 to 74024603; chromosome 2, from 0 to 30493730; chromosome 3, from 16493431 to 50407653; chromosome 4, from 7406888 to 50551374; chromosome 5, from 9881466 to 58473554; chromosome 6, from 3861081 to 33077717; chromosome 7, from 4056987 to 58629226; chromosome 8, from 4670213 to 54625578; chromosome 9, from 6225214 to 63773642; chromosome 10, from 3775719 to 55840828; chromosome 11, from 10947270 to 48379978; and chromosome 12, from 5879033 to 61255621.

### Principal coordinate analysis and genetic structure, diversity, and linkage disequilibrium

The genetic structure and the division in subpopulations were determined by conducting a series of hierarchical principal coordinate analyses (PCoAs). The PCoAs were carried out with a subset of the variants after filtering. The variant filtering process consisted of several steps. First, only the euchromatic variants were considered, and from those, only the ones with a call rate lower than 0.95 were selected. From this set of variants, the ones in which the minor allele was present in fewer than three samples were removed. For the remaining variants, 2000 evenly distributed across the genome were selected. Furthermore, to avoid overrepresentation of large regions in complete linkage disequilibrium (LD), when several consecutive variants had a correlation higher than 0.95, only one of them was kept. Finally, pairwise distances between samples were calculated (Kosman and Leonard, 2005). PCoA (Krzanowski, 2000) was generated from the distance matrix, following the pycogent implementation. These methods were implemented in the do_pca.py script. Additionally, the genetic structure was also estimated with fastSTRUCTURE (Raj *et al.*, 2014).

The observed and expected heterozygosity and the number of variants per genetic group were calculated considering only the variants variable in the samples involved in the analysis. The script that implemented these analyses is calc_diversities2.py. The allele spectrum figure was plotted by the script calc_maf_trends.py and the rarefaction curves by rarefaction_analysis.py.

The LD was calculated between euchromatic markers with a major allele frequency lower than 0.98 following the Rogers and Huff method for loci with unknown phase (Rogers and Huff, 2009).

### Detection of introgressions

To find introgressions typical of modern cultivars in the traditional varieties, a haplotype analysis was performed. The genome was divided into windows and, in each one, the Kosman distances were calculated from the non-traditional samples to the haplotypes found in the traditional samples. When the analyzed non-traditional sample haplotype had a non-zero distance from any of the traditional ones, it was marked as distant from the traditional collection.

### Genome-wide association study and allele frequencies

A heatmap plot that represents the major allele frequency in each group was generated according to a dendrogram by the method implemented in the Python seaborn library (Waskom, 2021) and was plotted by the get_most_diverse_snps.py script. 

A genome-wide association study (GWAS) was carried out with the Genesys R package (Gogarten *et al.*, 2019) on the set of polymorphic variants (95% threshold). The quantitative characters were normalized by using the Box and Cox transformation implemented by the Python scipy library (Virtanen *et al.*, 2020). The character normality was checked with a qqplot plotted by the Python statsmodels library (Seabold and Perktold, 2010). The correction for genetic structure was calculated with a principal component analysis on the filtered variants implemented by the SNPRelate R library with a 0.3 LD threshold (Zheng *et al.*, 2012). The quantitative trait associations were tested with the Wald method, and the binomial ones by the Score method. To account for the multiple tests, a Bonferroni threshold was applied. The step-by-step implementation of the GWAS analysis can be analyzed in the gwas.py script.

### Genetic group distances

Nei and Dest (Peakall and Smouse, 2006, 2012) genetic distances among groups were calculated and compared. They were implemented by the Python variation library and the cacl_pop_dists.py script. From those distances both a neighbor-joining tree and a split network were calculated using SplitsTree (Huson and Bryant, 2006).

## Results

### High-throughput genotyping of a European traditional tomato collection

To genetically characterize traditional European tomatoes, a total of 1254 tomato accessions were used (Supplementary Table S1). This set, which included an extensive representation of the extant European traditional tomato variability, comprised 506 accessions from Spain, 305 from Italy, 203 from Greece, 96 from France, and 58 from other origins, as well as 25 modern commercial cultivars and 39 SP and 22 SLC accessions (the last two being of American origin). A total of 3700 million reads with a mean phred quality of 33.5 were obtained after GBS, providing an average of 2.9 million reads per sample. Out of these, 99.0% were successfully mapped to the tomato reference genome (v2.50), but only 55.9% were kept after applying the MAPQ filter with a threshold of 57. These reads were mostly properly paired (96.1%). From all genomic positions that comprise the reference genome, 0.79% had an average sequencing coverage per sample higher than 5X, 0.46% higher than 10X, and 0.21% higher than 20X. The complete sequencing and mapping statistics for all samples are available in Supplementary Table S3, and the number of positions per megabase with more than five reads in at least 90% of the samples is presented in Supplementary Fig. S1. Finally, 448 121 variants were called by freebayes and, after filtering them, a working dataset of 64 943 variants was created.

### Genetically defining true European traditional tomatoes and their relationship with American relatives

**Fig. 1.**
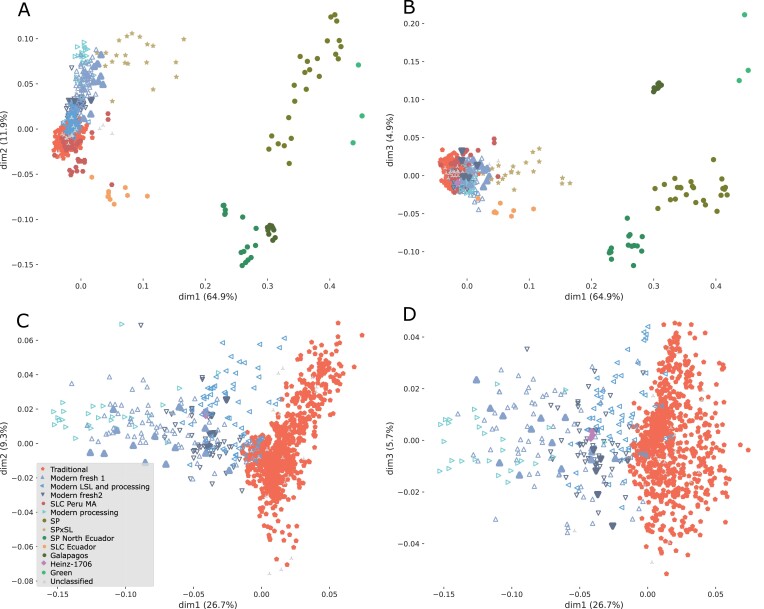
Principal coordinate analysis (PCoA) including cultivated tomato (*Solanum lycopersicum* var. *lycopersicum*; SLL): traditional European tomato, modern cultivars with different culinary use [fresh, processing, and long shelf-life (LSL)]; *S. lycopersicum* var. cerasiforme (SLC) from different origins [Peru, Mesoamerica (MA), Ecuador]; and several American wild relatives: *Solanum pimpinellifolium* (SP), *Solanum cheesmaniae*, *Solanum galapagense* (Galápagos), *Solanum peruvianum*, *Solanum chmielewskii*, *Solanum habrochaites* (Green), and SP×SL hybrids. The modern cultivar Heinz 1706 was included as reference. Empty symbols indicate accessions initially reported as traditional but reclassified as modern. (A) First and second principal components (dim1 and dim2) from the PCoA using all accessions analyzed in this study. (B) First and third components (dim1 and dim3) from the same PCoA. (C) First and second components (dim1 and dim2) from PCoA using only *S. lycopersicum* var. *lycopersicum* samples. (D) First and third components (dim1 and dim3) from the previous PCoA. The percentage of variance accounted for by each principal component is indicated on each axis.

**Fig. 2.**
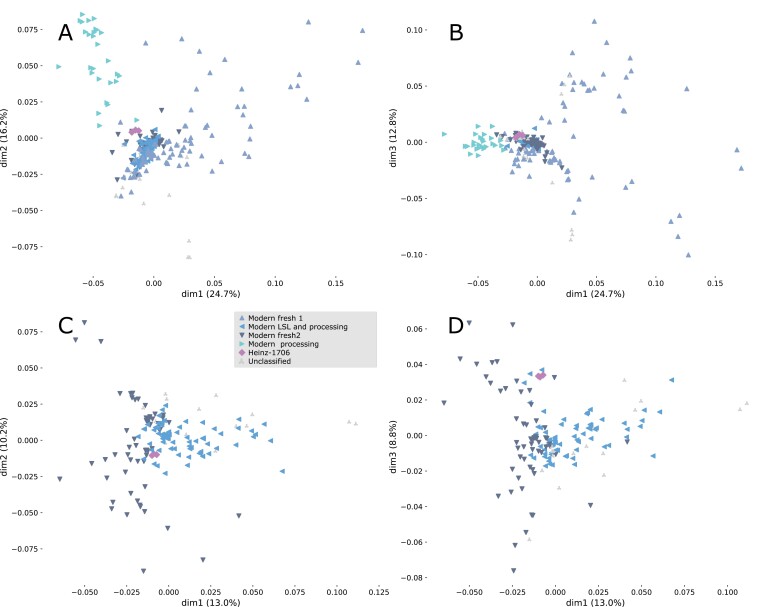
Principal coordinate analysis (PCoA) of modern tomato cultivars. (A, B) The three first principal components (dim1, dim2, and dim3) from the PCoA considering all modern cultivars and cv. Heinz 1706 as reference. (C, D) PCoA including only modern fresh 2 and long shelf-life (LSL) and modern processing genetic groups. The percentage of variance accounted for by each principal component is depicted on each axis.

To genetically position the European tomato collection relative to South American and Mesoamerican germplasms, which represent early domestication and improvement steps (Blanca *et al.*, 2015), the variability of European traditional tomatoes was analyzed together with SP, SLC, SL×SP hybrids, and a sample of modern cultivars. A series of PCoAs (Figs 1, 2) was performed comparing the traditional and modern collections. The genetic classification based on these PCoAs can be found in Supplementary Table S1 under the header rank1 classification.

The PCoA (Fig. 1A, B) showed that the green-fruited and Galapagos wild species, SP, SP_NEcu, Ecuadorian SLC (SLC_Ecu), Peruvian and Mesoamerican SLC (SLC_Peru_MA), and several SP×SL formed a series of clusters that were clearly separated from the modern and European traditional tomatoes (Fig. 1A, B). The SLC_Peru_MA group was the closest to the European traditional tomatoes. To obtain further insight into the genetic architecture of the European tomatoes, the genetic data were analyzed by using fastSTRUCTURE (Raj *et al.*, 2014). The model marginal likelihoods reached a plateau by four populations (Supplementary Fig. S2). By comparing with the PCoA classification, the four fastSTRUCTURE populations were found to correspond to SP, modern tomatoes, and two distinct traditional populations (Supplementary Fig. S2).

A continuous gradient from traditional to modern, rather than clearly split groups, was observed in the PCoA plots (Fig. 1A, C, D). To define the limits between modern and traditional in the PCoA, we chose Heinz 1706 as the reference (indicated in pink in Figs 1, 2), since it was one of the first tomato varieties reported to include introgressions from wild *Solanum* species on chromosomes 4, 9, 11, and 13 (Sato *et al.*, 2012; Causse *et al.*, 2013; Menda *et al.*, 2014), typical of modern cultivars carrying mainly disease resistance genes.

PCoA-based classifications indicated that a total of 24.9% of the accessions labelled as traditional according to their passport data mapped outside the traditional genetic cluster but within the modern and SP×SL genetic groups (Fig. 1) Several accessions initially catalogued as traditional that mapped close to the modern accessions in the PCoA space were consistently found to include haplotypes not present in the traditional group (Supplementary Fig. S3) and thus were reclassified as modern accessions (Supplementary Table S1).

The modern accessions (including both modern references and the traditional accessions reclassified as modern) were spread across the PCoAs according to their use (fresh or processing) and their degree of introgression (Figs 1C, D, 2; Supplementary Fig. S3). PCoAs applied only to the modern accessions resulted in four groups (Fig. 2): modern processing (the most distant group to Heinz 1706), modern long shelf-life (LSL) and processing (the closet to Heinz 1706), modern fresh 1 (between modern processing and Heinz 1706), and modern fresh 2 (closer to Heinz 1706). Each group was characterized by the presence of different introgressions in several chromosomes (Supplementary Fig. S3).

### Diversity among European traditional tomatoes

European traditional tomatoes are usually considered to have low genetic diversity (Blanca *et al.*, 2015). Therefore, it was important to calculate the number of polymorphic variants present in our collection of European traditional tomatoes, the largest collection analyzed by sequencing thus far, and to compare it with the variability present in the wild SP, the wild and semi-domesticated SLC, and the modern cultivars. The total number of variants within the European traditional collection was 26 129, larger than the number found in SP (19 164), SLC (7690), or the materials classified as modern (17 328). However, this comparison could be biased in favor of the traditional collection because of the larger number of traditional samples (890) compared with SP (24), SLC (42), and modern (243).

**Fig. 3.**
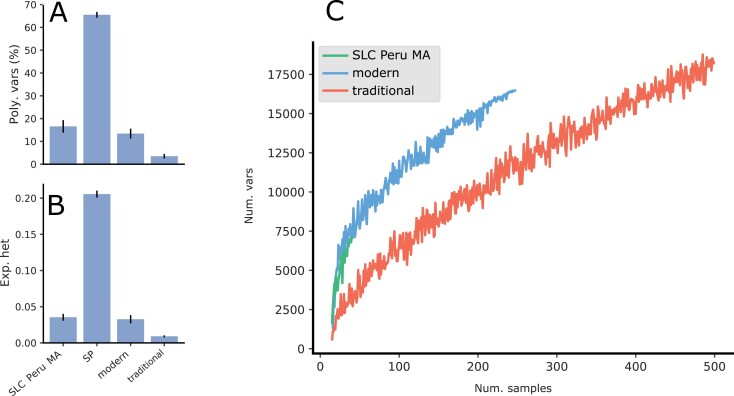
Genetic diversity for the rank1 genetic groups. (A, B) Genetic diversity estimated by (A) the percentage of polymorphic variants and (B) the expected heterozygosity, both calculated after applying the 95% threshold. The indices were calculated 100 times, taking 20 samples at random from each genetic group. The mean and SD are shown. (C) Rarefaction analysis of the number of variants found in each genetic group. The x-axis shows the number of samples and the y-axis shows the number of variants.

To correct for this factor, diversity indices were calculated with the same number of samples (20) (Fig. 3A, B) and the analysis was repeated 100 times with a different set of 20 samples chosen at random each time. Both the Nei diversity and the percentage of polymorphic variants (with a 95% threshold) was much higher in the wild SP than in any other group, and both indices were the lowest, by far, in the traditional group. The analysis indicated that many of the variants found in the traditional collection could not be considered polymorphic. At a 95% threshold, the traditional collection contained only 298 polymorphic variants. This scarcity in polymorphic variants in the European traditional group can also be observed in the allele frequency spectrum (Supplementary Fig. S4), in which it is clear that most variants in the traditional collection were almost fixed.

To better compare the amount of genetic variability in each major cultivated group (SLC_Peru_MA, traditional, and modern) a rarefaction analysis was carried out (Fig. 3C). The number of variants found in the traditional group was always lower than in the modern and SLC_Peru_MA groups, but the total number of variants within the traditional collection kept increasing as more samples were added. However, the number of polymorphic variants stabilized with a few samples. Conversely, the Nei diversity decreased (Supplementary Fig. S5) when more samples were added. This decrease was due to the high number of variants found within the traditional group that were close to fixation.

### Linkage disequilibrium

The LD was calculated for SP, SLC from Peru and Mesoamerica, modern, and European traditional varieties, those genetic groups with enough polymorphic markers [minimum allele frequency (MAF) >0.02 threshold] (Supplementary Fig. S6). The wild SP showed the lowest LD (*r*^2^=0.42) at 5 kb and was the group in which LD decreased the fastest, being only *r*^2^=0.2 at 25 kb. In SLC, *r*^2^ was 0.8 at 5 kb and 0.4 at 1000 kb. Traditional tomatoes had the highest LD at 25 kb (*r*^2^=0.97); however, it decreased to the lowest value (*r*^2^=0.05) at 1000 kb. The modern accessions had a high LD both at 25 kb (*r*^2^=0.9) and at 1000 kb (*r*^2^=0.6). The LD found at 1000k kb is likely due to population substructure. The SLC and modern groups had high long-range LDs, perhaps because the modern group included both fresh and processing accessions, which were clearly separated in the PCoAs, and SLC contained accessions from Peru and Mesoamerica, two geographically distant areas. Additionally, modern cultivars often contain introgressions from wild species, including disease resistance genes, that span large regions for which recombination is usually suppressed. SP is also known to have a clear population structure (Blanca *et al.*, 2012) and also showed some long-range LD, which clearly supports the conclusion that LD is due not just to gamete disequilibrium, but to other causes too. The traditional accessions showed the lowest LD at 1000 kb, perhaps because this group has a less remarkable population substructure.

### Classification of traditional tomato clusters

To further classify true traditional tomatoes, a series of PCoAs (Supplementary Fig. S7) was performed. A genetic group was created when several samples that grouped together in the PCoAs shared their geographic origin or traditional variety name, or some aspect of their phenotype (Supplementary Table S2), for example, fruit shape and size. Most traditional samples could be classified into 27 different genetic groups by using this PCoA strategy (Supplementary Table S2). The genetic classification of traditional tomatoes based on these PCoAs can be found in Supplementary Table S1 under the header rank2 classification.

**Fig. 4.**
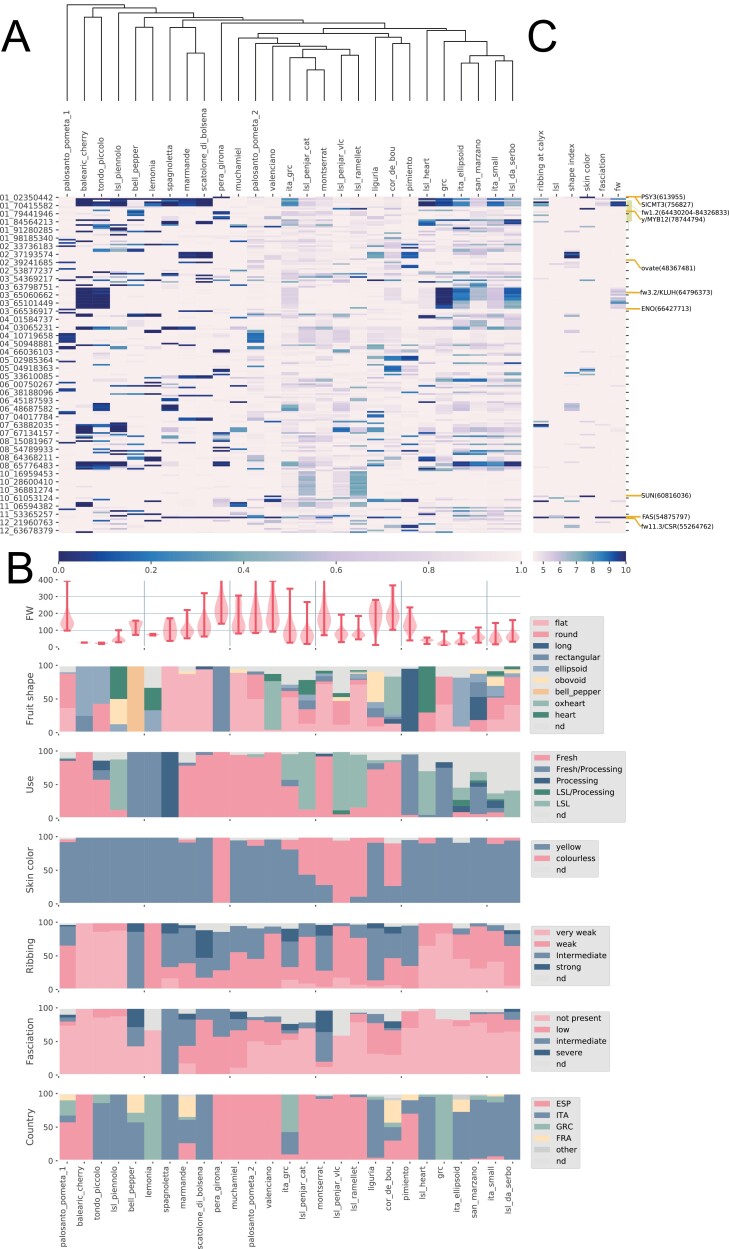
Allele frequencies across the genome in traditional genetic groups and their relationship with phenotypic diversity. (A) Clustering of genetic groups based on allele frequencies. The allele frequency of the major allele within each genetic group is indicated by a color density according to the legend (blue, frequency=0, to white, frequency=1. (B) Distribution of the different traits within genetic groups. (C) Statistical significance indicated by a colored gradient of –log(*P*) values of the SNP–trait associations by GWAS. The positions of candidate QTLs and genes are depicted to the right of the association columns.

Two connected clusters of genetic groups (for the sake of clarity we will use ‘group’ to refer to a PCoA group of samples and ‘cluster’ to describe a cluster of groups) were observed in PCoA (Supplementary Fig. S7A, B). Within the cluster at the center of PCoA, we found seven Spanish genetic groups, two Italian, and one Greek (Supplementary Fig. S7C, H), together with ‘Marmande’, ‘Bell pepper’ and ‘Palosanto pometa 1’ (Fig. 4A), which were represented in all four Mediterranean countries (Spain, Italy, France, and Greece). Outside the central cluster, but close to it, we found groups of large tomatoes from all four countries (Supplementary Fig. S7C, D; Fig. 4A, B). A second cluster included mostly Italian accessions and some Greek and Spanish accessions, all characterized by having a small size with no or weak ribbing (Supplementary Fig. S7A, B, M–R; Fig. 4A, B). In summary, the PCoA separated traditional accessions mainly by country of origin and fruit size. It is interesting to note that the LSL-type accessions, which were highly represented in the collection, were not grouped together, but rather segregated by country: the Italian LSL varieties were found within the Italian cluster, and the Spanish LSL within the Spanish cluster. Several accessions located between the Spanish and Italian clusters could not be grouped by passport data or any other characteristic.

### Allele frequencies across the genome in traditional groups and their relationship with phenotypic diversity

The clustering of the traditional genetic groups based on a distance tree was calculated using the polymorphic variants (Fig. 4A). The defined genetic groups had quite distinct allele frequencies along the genome. Concomitantly, the genetic groups also showed enrichment in specific phenotypic characteristics related to their horticultural classification (Fig. 4B). Furthermore, several noticeable clusters of genetic groups with common phenotypic traits could be observed. For instance, there was a cluster formed by small-fruited, slightly ribbed, LSL and processing Italian genetic groups, which included the well-known Italian ‘*da Serbo*’ and ‘*San Marzano*’ tomatoes. Another cluster was constituted mainly of LSL colourless-skinned Spanish tomatoes. Close to this cluster were some of the most typical Spanish traditional fresh-market varieties.

Genetic differences between the groups could be due to genetic drift or either inadvertent or conscious selection by traditional farmers. In order to elucidate whether the differentiating variants were associated with the phenotypic variation, a GWAS analysis was carried out using selected fruit traits (Fig. 4C).

Two of the main phenotypic characteristics differentiating the traditional tomatoes are fruit weight (*fw*) and ribbing (Fig. 4B). In the GWAS analysis, fruit weight was associated with variants on chromosomes 1, 3, and 11. The MAF analysis indicated that most of the small-fruited tomatoes shared the fixation of the same allelic variant in chromosome 1. The pattern found in chromosome 3 was similar. The position of candidate quantitative trait loci (QTLs) and genes is depicted to the right of the association columns in Fig. 4C. In contrast, almost all medium and large tomatoes had a fixed common variant in chromosome 11 that was associated by GWAS with fruit weight, ribbing at the calyx end, and fruit shape index.

Another trait differentiating traditional tomato cultivars was skin colour; for instance, most Spanish LSL tomatoes as well as tomatoes included in the ‘Cor de bou’, ‘Montserrat’, and ‘Pera girona’ genetic groups had colourless skin, which resulted in pinkish fruit (Fig. 4B). GWAS found associations with pink color in chromosomes 1 (two regions), 3, 5, and 10. Comparison of the GWAS and MAF analyses (Fig. 4A, C) showed that different pink genetic groups had different allelic compositions in the associated genomic regions, which might reflect a complex genetic control.

Fruit shape was associated with regions in chromosomes 2, 5, 10, and 12. The region in chromosome 2 was fixed in ‘Marmande’ and ‘Scatolone di bolsena’, two groups that are well known for having flat fruits. High-frequency minor alleles, almost fixed in the regions associated with fruit shape in the GWAS, were also observed in other genetic groups, such as the Italian ‘LSL da Serbo’, in chromosome 5, and ‘Ita ellipsoid’ and ‘Tondo Piccolo’, in chromosome 6, as well as in the ‘Cor de bou’ and *‘*Pimiento’ groups, in chromosome 12.

In the case of use, associations were found in chromosomes 10 and 11, but, in this case, no clear relationship was found between allelic frequencies among the tomato genetic groups and GWAS.

### Network analyses support the differentiation between Spanish and Italian traditional tomatoes and the occurrence of hybridization events in traditional tomatoes across Europe

To study the genetic relationships between accessions and groups of accessions, a network based on pairwise Dest group distances was created with SplitsTree. Evolutionary relationships are often represented as a unique tree under the assumption that evolution is a branching or tree-like process (Huson, 1998). However, real data do not always clearly support a tree. Phylogenetic split decompositions represented in a network may be evidence for conflicting reticulated phylogenies due to gene flow and/or hybridization (Huson, 1998).

**Fig. 5.**
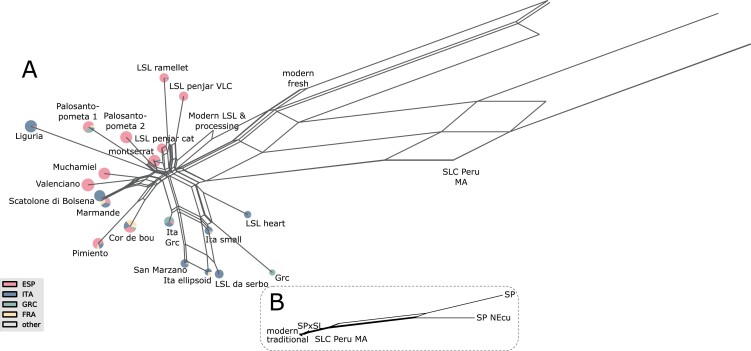
Evolutionary relationships between traditional European tomato, modern tomato, and Peru and Mesoamerica *Solanum lycopersicum* var. *cerasiforme* (SLC), *Solanum pimpinellifolium* (SP), and SP×SL hybrids. Split network based on the Dest distances between genetic groups. The country of origin of accessions within each genetic group is represented by a pie chart (colors are defined in the key shown in the bottom left). (A) Zoom only on European modern and traditional tomatoes. (B) Zoom on American ancestral and wild tomatoes. Each edge of the network represents a split of the accessions based on one or more characteristics. If there was no conflict, each split is represented by a single edge, whereas in the case of contradictory patterns the partition is represented by a set of parallel edges. The edge lengths represent the weight of each split, which is equivalent to the distance between groups. ESP, Spain; GRC, Greece; FRA, France; ITA, Italy; LSL, long shelf-life; MA, Mesoamerica; NEcu, North Ecuador; VLC, Valencia (Spain).

The SplitsTree network of European tomato is depicted in Fig. 5. Like in the PCoAs (Supplementary Fig. S7), the group organization in the network (Fig. 5) was structured in two main country-related clusters. One cluster was composed mainly of Spanish traditional groups, which included the Spanish LSL, and the other cluster was composed mostly of the small-fruited Italian LSL and processing groups. Interestingly, the ‘Liguria’ group, composed mainly of Italian accessions (Supplementary Table S1), clustered with Spanish clusters, although the branch that linked it with the core Spanish clusters was quite large.

The degree of reticulation found (Fig. 5) suggested that hybridizations might have occurred between the ancestors of accessions collected from the same geographic regions. On the other hand, the groups that included accessions from different countries, such as ‘Marmande’, ‘Pimiento’, ‘Cor de bou’, or ‘Palosanto pometa 1’, were located between the Spanish and Italian clusters.

These groups of mixed origin could be more modern and derived from hybridization from older Spanish and Italian varieties or, alternatively, they could be very old varieties found across Europe before the Spanish and the Italian diversification started. To test these possibilities, a rarefaction analysis was performed of the number of polymorphic sites found in these three clusters (Supplementary Fig. S8). The number of polymorphic sites was clearly higher in the Italian and Spanish clusters and much lower in the mixed-origin cluster, evidence that supports that Spain and Italy were secondary centers of diversity for the European tomato, whereas the varieties included in the mixed cluster would be more recent.

## Discussion

### Very low, but discriminant, variation in traditional European tomatoes

The genetic diversity of the European traditional collection was very low compared with the diversity found in SP or SLC, in agreement with previous surveys on worldwide SLL accessions (Blanca *et al*., 2012, 2015; Sim *et al.*, 2012a). Nevertheless, the current analysis represents the first estimate obtained using a comprehensive representation of traditional European tomatoes, and it is relevant to study the role of Europe as a secondary center for tomato diversification. The low level of diversity found in these traditional materials was quite striking and remarkable when we consider the high phenotypic diversity of traditional tomatoes. Moreover, the high LD found in the traditional varieties suggests that it is rather unlikely that the total number of polymorphic blocks would grow much even if whole genome sequences were to be obtained.

Previous studies demonstrated a strong bottleneck during the SLC tomato’s travel from Ecuador and Peru to Mesoamerica (Lin *et al.*, 2014; Blanca *et al.*, 2015; Razifard *et al.*, 2020). However, despite the low genetic diversity found in traditional European tomatoes, there are still a few highly polymorphic loci within this gene pool. Some of this variation could be due to the random nature of genetic drift. However, the association study revealed that a sizeable fraction of those diverse loci was associated with the traditional fruit phenotypic/morphological variation. Therefore, it is quite likely that many of those polymorphic loci had been under balancing selection (Delph and Kelly, 2014) during the diversification process and were, in fact, responsible for a sizeable part of the tomato phenotypic variation or, at least, in LD with the variants selected. It may seem paradoxical that the high diversity of shapes, colors, sizes, uses, and other agronomic traits in the traditional group could be maintained by such a poor gene pool, but it seems that the selection carried out by the traditional growers in favor of this agronomic diversity resulted in a desert of variation, with just a handful of scattered polymorphic loci. This is consistent with two highly polymorphic SNPs found in the *lc* locus (Muños *et al.*, 2011) surrounded by loci with ‘drastically reduced’ diversity. Thus, the high polymorphism seemed to be the result of divergent selection for a low or high number of locules in different cultivars.

Recently, structural variants were studied in tomato using new long-read sequencing technologies and new analysis algorithms (Alonge *et al.*, 2020; Domínguez *et al.*, 2020). A large number of structural variants were identified and were mostly generated by transposons and related repeats. Similar to the variants studied here, most structural variants had a very low frequency, and the majority were singletons. Therefore, the phenotypic diversity present in European traditional tomatoes seems to have been built by remixing, reshuffling, and swapping very few polymorphisms with the selection pressure associated with the creation of new varietal types and the adaptation of these types to different regional environments.

### History of tomato movement in Europe

The distribution of the genetic variability in the European traditional tomatoes showed mostly a continuous gradient. However, the Spanish and Italian varieties occupied opposite regions of the PCoA space, which supports a genetic differentiation among varieties originating in those countries. The lack of clear-cut limits might be due to the limited resolution of the current GBS analysis; resequencing of the traditional varieties would provide further details. Nevertheless, we do not expect a different general conclusion given the high LD found among the European traditional tomatoes. Additionally, Spain and Italy share a long common history, which also supports that the lack of limits would be due to migrations between different regions and countries and subsequent intercrossing. Despite this difficulty, the genetic traditional groups proposed here were differentiated by characteristics such as their main geographic origin, use, fruit morphology, and varietal name. The genetic groups sometimes corresponded with the varietal type (e.g. ‘Valenciano’, ‘Muchamiel’, ‘Penjar’). However, the match between the proposed genetic group and the sample varietal name was seldom complete (the ‘Cor de bou’ group included two ‘Valenciano’, one ‘Russe’, and one ‘Costoluto’ samples). This may be due to the limitations of the genetic classification methodology utilized or to erroneous passport data. Other genetic groups, such as ‘Italian small’, showed no clear associations with any varietal name. Finally, cultivars previously classified as belonging to the same variety, such as ‘Marmande’, were included in many different genetic groups. It is likely that the popularity of some varietal types, such as ‘Marmande’ (typical large and multi-locule tomatoes), made some growers prone to apply the label to any variety that displayed the typical morphological characteristics of a well-known varietal type.

One clear example of mistaken identity and/or inadvertent outcrossing is provided by some supposed traditional varieties that we reclassified as modern, which may either have been misclassified or correspond to a mixture between traditional and modern varieties. It is not trivial to define the borderline between traditional and modern varieties. One could think that until the 1950s most varieties were heirlooms and landraces maintained by small farmers, but the real history is more complex. When tomato cultivation was popularized in the 19th century in France, England, and the USA, some of the varieties were already provided by seed companies (Boswell, 1937). Seed shipments are documented between countries, for instance, from England to the Canary Islands (Amador *et al.*, 2013). Moreover, from 1910 onwards, professional breeding efforts created new varieties adapted to long-distance shipping and for processing (Boswell, 1937). These efforts did not yet include wild materials, so the genetic diversity of early-bred accessions is not easy to differentiate from traditional diversity in a PCoA analysis. It was only after breeders started introgressing alleles from wild species for disease resistance that the genetic diversity was different enough to be easily differentiated in the PCoA analysis. In any case, the traditional–modern distinction has to be somewhat conventional, although a characteristic of modern cultivars compared with traditional varieties is the introgression of genes from wild species. Therefore, true traditional cultivars were defined based on the absence of wild species’ haplotypes. Cultivars carrying any of those introgressions came from modern breeding programs or were the result of a cross with modern cultivars.

Most of these introgressions seem to be related to disease resistance genes such as the *Cladosporium fulvum* resistance gene *Cf-2* on chromosome 6 and *Tm-2* (resistance to tomato mosaic virus) on chromosome 9. It is likely that the modern genetic variability has been combined with the true traditional varieties, so that some materials catalogued in the gene banks as ‘traditional’ are in fact a mixture of traditional and modern. This is to be expected, as the seed collectors/gene banks label as ‘traditional’ any material considered as such by the farmer from whom the seeds were collected. Although European small farmers often save their own tomato seeds, they may occasionally purchase or obtain plantlets from markets or nurseries, or save seeds from modern varieties purchased in the market and introduce them into their fields. This may lead, after several years of reproduction and farmer selection, to complex hybridizations and mixings. Clearly, there have been many opportunities for introgressing modern haplotypes into the traditional materials, such as unintentional crosses. This leakage might have a positive unintended consequence of increasing the very low diversity of the traditional pool, and it is also the case that evolution consists of change and adaptation of local varieties (Casañas *et al.*, 2017). Therefore, traditional and modern varieties have not been isolated, and genetic exchange has occurred between them from the early professional breeding to the present day. This genetic exchange may explain the continuous genetic gradient and the lack of clear split between modern and traditional varieties. The haplotypes described herein could be used for the identification of non-true European traditional tomatoes.

The allele-frequency-based tree (Fig. 4A) defined three major clusters: Spanish, Italian, and mixed origin. The mixed-origin groups were basal in the tree in Fig. 4A, have longer branch lengths, and occupy an intermediate position between the Italian and Spanish clusters in the Dest network (Fig. 5). These results are compatible with the hypothesis that Italy and Spain formed two centers of diversity. The differentiation of Italian and Spanish gene pools is exemplified by the LSL varieties from both countries. Italian and Spanish LSL varieties were clustered apart from each other, with only a small number of samples from the other country. So, it seems as if the origin of the LSL tomatoes in both countries was independent. The transformation from a fresh to an LSL variety is likely due to a limited number of loci, as observed in Fig. 4A, in which the Catalonian fresh ‘Montserrat’ type is closely related to the Catalonian LSL ‘Penjar’ type. Esposito *et al.* (2020) also observed geographic differentiation of the Italian and Spanish LSL varieties. Therefore, although there may have been migrations from Italy to Spain and vice versa, these may not have been extensive enough to erase the genetic differences between the Italian and Spanish varieties.

Regarding the mixed-origin cluster, the groups included in it were basal in the tree shown in Fig. 4A, have longer branch lengths, and occupy an intermediate position between the Italian and Spanish clusters in the Dest network (Fig. 5). Moreover, the rarefaction analysis supports that this cluster included varieties derived from the two secondary centers of diversity. This could be the result of the long tradition of tomato cultivation in Southern Europe, with the groups included in this cluster being developed from hybridizations between the two centers of diversity (i.e. Spain and Italy). New mutations, other introductions of tomatoes from America, or new genes from varieties developed worldwide might also be involved in the history of the groups of mixed origin.

A complex pattern of migrations can also be inferred in several genetic groups, such as the ‘Cor de bou’ group, which included varieties from France, Italy, and Spain. The Italian ‘Spagnoletta’ group was closely related to the ‘Marmande’ group constituted of French, Spanish, Greek, and Italian accessions. Other genetic groups with mixed geographic origin are ‘Liguria’, ‘Pimiento’, and ‘Palosanto Pometa 1’.

### Do a few polymorphic genes differentiate the true European traditional tomato genetic groups?

In order to shed light on the apparent contradiction between the low genetic diversity and the large phenotypic variation of European traditional tomatoes, a GWAS was carried out with the polymorphic variants and some of the most obvious morphological traits (fruit morphology, color, and ripening behavior). Variants located in the genomic regions of previously identified loci involved in fruit weight, and likely involved in domestication and diversification, were associated with this trait in the GWAS. Most of the small-fruited tomatoes shared fixed variation regions in chromosomes 1 and 3, which mapped close to previously described QTLs and genes associated with fruit size (Fig. 4A, B): *fw1.2* (Grandillo *et al.*, 1999) and *fw3.2/KLUH* and *ENO* (Chakrabarti *et al.*, 2013; Yuste-Lisbona *et al.*, 2020). In contrast, almost all medium and large tomatoes shared a region in chromosome 11 that mapped close to *FAS* (Xu *et al.*, 2015) and *fw11.3*/*CSR* (Mu *et al.*, 2017), with both genes playing a known role in controlling fruit size and fasciation. No further associations were observed in other genomic regions for fruit weight, so it seems reasonable to think that these QTLs might be responsible, at least in part, for the variability in fruit size among the European traditional tomatoes. Regarding fruit shape, the associated region on chromosome 2 includes *ovate* (Liu *et al.*, 2002) and other fruit-shape QTLs (Brewer *et al.*, 2007), and the region on chromosome 10 is located close to the position where the original copy of the *sun* locus was found (Xiao *et al.*, 2008). In the case of skin color, a different pattern was characteristic of different pink genetic groups. ‘*LSL Penjar* vlc’ and ‘*LSL ramellet*’ shared a variant at the end of chromosome 1 that matched a region that was previously associated with skin color, the colorless-peel *y-*locus (Ballester *et al.*, 2010), while ‘*Pera Girona*’ had the minor allele for the other chromosome 1 variant, which is located at the beginning of the chromosome and maps close to the *SlCMT3* (Gallusci *et al.*, 2016) and *PSY3* (Li *et al.*, 2008) genes, involved in epigenetic ripening regulation and carotene biosynthesis, respectively.

The current analysis suggests that variability in fruit morphology among European traditional tomatoes could be the consequence of the combination of a relatively low number of genes, as suggested by Rodríguez *et al.*, (2011), including *fw3.2/KLUH*, *ENO*, *FAS*, *SUN*, and *OVATE*. On the other hand, skin color could be a consequence of *y*-locus and other genes related to phenylpropanoid metabolism. Interestingly, structural variant mutations have been found in *fw3.2/KLUH*, *FAS*, and *SUN*, which supports the impact of structural variants on tomato phenotypic diversity (Alonge *et al.*, 2020; Domínguez *et al.*, 2020). In addition, some cryptic variation hidden in the Mesoamerican tomatoes may have emerged in European tomatoes after generating new combinations and divergent selection by the traditional farmers, as found for the jointless trait in tomato (Soyk *et al*., 2017, 2019; Alonge *et al.*, 2020).

### Impact on gene bank and on-farm variability management

Many of the few polymorphic genetic variants within the very-low-diversity European traditional tomatoes appeared to be associated with phenotypic variation. This has implications for the conservation efforts carried out by gene banks. Thousands of European traditional tomatoes are maintained in many of these gene banks. However, the cost of these conservation efforts could be drastically reduced if only these few polymorphic loci were considered. Of course, such an approach would ignore most variants, the ones found in very low frequencies, but conserving these low-frequency alleles, which in many cases would be neutral and thus not associated with any phenotypic variation, requires a sizeable investment. An alternative would be to identify the alleles associated with a phenotype; however, this would require an exhaustive phenotypic characterization.

Most of the European accessions analyzed here were collected from farmers during the 1950s to the 1980s and, as landraces, they are appreciated, competitive, and cultivated varieties. The genetic diversity of many other crops has also been maintained as landraces that evolved on-farm. However, this diversity is continuously under threat by the introduction of new modern varieties derived from a limited gene pool that have replaced the traditional varieties. It is generally believed that most of the accessions in seed banks do not contribute to modern varieties (Tanksley and McCouch, 1997), and this is also the case for tomato. Our identification of the morphological and genetic structure present in the European traditional tomato gene pool will be important to guarantee access to that variability as the basis for the development of new varieties or evolved landraces in the future (Casañas *et al.*, 2017).

### Conclusion

The entrepreneurship of many local European farmers during the past 500 years has managed to create a very complex and diverse set of tomato varieties adapted to different local tastes and morphological preferences. These localized activities did not restrain farmers from importing other interesting novelties developed by other farmers elsewhere, thus generating a much larger set of varietal tomato types that are characterized by an exuberant diversity that serves as a variety for fresh, processing, and LSL uses. The current report shows that such a plethora of different types has been created from an original material devoid of genetic diversity, by exploiting very few polymorphic loci subjected to balancing selection.

## Supplementary data

The following supplementary data are available at *JXB* online.

Fig. S1. Number of genomic positions with high coverage and number of variants per Mb along the genome in all accessions.

Fig. S2. FastSTRUCTURE analysis.

Fig. S3. Introgressed regions along the genome detected in the modern genetic groups.

Fig. S4. Major allele frequency spectrum in the traditional, modern, and SLC_Peru_MA groups.

Fig. S5. Rarefaction analysis of the expected heterozygosity for each genetic group.

Fig. S6. Genome-wide LD decay in wild, SLC, traditional, and modern accession groups.

Fig. S7. Hierarchical PCoA of European traditional tomato varieties.

Fig. S8. Rarefaction analysis of the number of polymorphic variants (95% threshold).

Table S1. Accessions analyzed in this study.

Table S2. Phenotypic characterization of European traditional tomatoes.

Table S3. Sequencing and mapping statistics for each sample.

erac072_suppl_Supplementary_Material_1

erac072_suppl_Supplementary_Material_2

## Data Availability

The sequence data can be found in NCBI (https://www.ncbi.nlm.nih.gov/sra) database under accession number PRJNA722111. All other data supporting the findings of this study are available within the paper and within its supplementary data published online

## Abbreviations

GBS: Genotyping by sequencing
GWAS: genome-wide association study
LD: linkage disequilibrium
LSL: long shelf-life
MAF: minimum allele frequency
PCoA: principal coordinate analyses
QTL: quantitative trait locus
SLL: *Solanum lycopersicum* L. var. *lycopersicum*
SLC: *Solanum lycopersicum* var. *cerasiforme*
SNP: single nucleotide polymorphism
SP: *Solanum pimpinellifolium*

## References

[CIT0001] Alonge M , Wang X, Benoit M, et al. 2020. Major impacts of widespread structural variation on gene expression and crop improvement in tomato. Cell 182, 145–161.e23. 32553272 10.1016/j.cell.2020.05.021 PMC7354227

[CIT0002] Amador LJ , Santos Coello B, Ríos Mesa D. 2013. Variedades tradicionales de tomates de canarias. Santa Cruz de Tenerife: CULTESA.

[CIT0003] Baird NA , Etter PD, Atwood TS, Currey MC, Shiver AL, Lewis ZA, Selker EU, Cresko WA, Johnson EA. 2008. Rapid SNP discovery and genetic mapping using sequenced RAD markers. PLoS One 3, e3376. 18852878 10.1371/journal.pone.0003376 PMC2557064

[CIT0004] Ballester AR , Molthoff J, de Vos R, et al. 2010. Biochemical and molecular analysis of pink tomatoes: deregulated expression of the gene encoding transcription factor SlMYB12 leads to pink tomato fruit color. Plant Physiology 152, 71–84. 19906891 10.1104/pp.109.147322 PMC2799347

[CIT0005] Bauchet G , Causse M. 2012. Genetic diversity in tomato (*Solanum lycopersicum*) and its wild relatives. In: Çalişkan M, ed. Genetic diversity in plants. London: IntechOpen.

[CIT0006] Blanca J , Cañizares J, Cordero L, Pascual L, Diez MJ, Nuez F. 2012. Variation revealed by SNP genotyping and morphology provides insight into the origin of the tomato. PLoS One 7, e48198. 23118951 10.1371/journal.pone.0048198 PMC3485194

[CIT0007] Blanca J , Montero-Pau J, Sauvage C, Bauchet G, Illa E, Díez MJ, Francis D, Causse M, van der Knaap E, Cañizares J. 2015. Genomic variation in tomato, from wild ancestors to contemporary breeding accessions. BMC Genomics 16, 257. 25880392 10.1186/s12864-015-1444-1 PMC4404671

[CIT0008] Boswell VR. 1937. Improvement and genetics of tomatoes, peppers, and eggplant. In: Yearbook of the United States Department of Agriculture. Washington, DC: United States Government Printing Office, 176–206.

[CIT0009] Brewer MT , Moyseenko JB, Monforte AJ, van der Knaap E. 2007. Morphological variation in tomato: a comprehensive study of quantitative trait loci controlling fruit shape and development. Journal of Experimental Botany 58, 1339–1349. 17283371 10.1093/jxb/erl301

[CIT0010] Casals J , Rivera A, Sabaté J, Romero del Castillo R, Simó J. 2019. Cherry and fresh market tomatoes: differences in chemical, morphological, and sensory traits and their implications for consumer acceptance. Agronomy 9, 9.

[CIT0011] Casañas F , Simó J, Casals J, Prohens J. 2017. Toward an evolved concept of landrace. Frontiers in Plant Science 8, 145. 28228769 10.3389/fpls.2017.00145 PMC5296298

[CIT0012] Causse M , Desplat N, Pascual L, et al. 2013. Whole genome resequencing in tomato reveals variation associated with introgression and breeding events. BMC Genomics 14, 791. 24228636 10.1186/1471-2164-14-791 PMC4046683

[CIT0013] Chakrabarti M , Zhang N, Sauvage C, et al. 2013. A cytochrome P450 regulates a domestication trait in cultivated tomato. Proceedings of the National Academy of Sciences, USA 110, 17125–17130. 10.1073/pnas.1307313110 PMC3801035 24082112

[CIT0014] Delph LF , Kelly JK. 2014. On the importance of balancing selection in plants. New Phytologist 201, 45–56. 23952298 10.1111/nph.12441 PMC3886833

[CIT0015] Domínguez M , Dugas E, Benchouaia M, Leduque B, Jiménez-Gómez JM, Colot V, Quadrana L. 2020. The impact of transposable elements on tomato diversity. Nature Communications 11, 4058. 10.1038/s41467-020-17874-2 PMC7426864 32792480

[CIT0016] Elshire RJ , Glaubitz JC, Sun Q, Poland JA, Kawamoto K, Buckler ES, Mitchell SE. 2011. A robust, simple genotyping-by-sequencing (GBS) approach for high diversity species. PLoS One 6, e19379. 21573248 10.1371/journal.pone.0019379 PMC3087801

[CIT0017] Esposito S , Cardi T, Campanelli G, Sestili S, Díez MJ, Soler S, Prohens J, Tripodi P. 2020. ddRAD sequencing-based genotyping for population structure analysis in cultivated tomato provides new insights into the genomic diversity of Mediterranean ‘da serbo’ type long shelf-life germplasm. Horticulture Research 7, 134. 32922806 10.1038/s41438-020-00353-6 PMC7459340

[CIT0018] Gallusci P , Hodgman C, Teyssier E, Seymour GB. 2016. DNA methylation and chromatin regulation during fleshy fruit development and ripening. Frontiers in Plant Science 7, 807. 27379113 10.3389/fpls.2016.00807 PMC4905957

[CIT0019] García-Martínez S , Andreani L, Garcia-Gusano M, Geuna F, Ruiz JJ. 2006. Evaluation of amplified fragment length polymorphism and simple sequence repeats for tomato germplasm fingerprinting: utility for grouping closely related traditional cultivars. Genome 49, 648–656. 16936844 10.1139/g06-016

[CIT0020] Garrison E , Marth G. 2012. Haplotype-based variant detection from short-read sequencing. arXiv 1207.3907. https://arxiv.org/abs/1207.3907. [Preprint].

[CIT0021] Gogarten SM , Sofer T, Chen H, Yu C, Brody JA, Thornton TA, Rice KM, Conomos MP. 2019. Genetic association testing using the GENESIS R/Bioconductor package. Bioinformatics 35, 5346–5348. 31329242 10.1093/bioinformatics/btz567 PMC7904076

[CIT0022] Grandillo S , Ku HM, Tanksley SD. 1999. Identifying the loci responsible for natural variation in fruit size and shape in tomato. Theoretical and Applied Genetics 99, 978–987.

[CIT0023] Harvey M , Quilley S, Beynon H. 2003. Exploring the tomato: transformations of nature, society, and economy. Cheltenham: Edward Elgar.

[CIT0024] Huson DH. 1998. SplitsTree: analyzing and visualizing evolutionary data. Bioinformatics 14, 68–73. 9520503 10.1093/bioinformatics/14.1.68

[CIT0025] Huson DH , Bryant D. 2006. Application of phylogenetic networks in evolutionary studies. Molecular Biology and Evolution 23, 254–267. 16221896 10.1093/molbev/msj030

[CIT0026] Kosman E , Leonard KJ. 2005. Similarity coefficients for molecular markers in studies of genetic relationships between individuals for haploid, diploid, and polyploid species. Molecular Ecology 14, 415–424. 15660934 10.1111/j.1365-294X.2005.02416.x

[CIT0027] Krzanowski WJ. 2000. Principles of multivariate analysis: a user’s perspective. New York: Oxford University Press.

[CIT0028] Li H. 2011. Improving SNP discovery by base alignment quality. Bioinformatics 27, 1157–1158. 21320865 10.1093/bioinformatics/btr076 PMC3072548

[CIT0029] Li H. 2013. Aligning sequence reads, clone sequences and assembly contigs with BWA-MEM. ArXiv 1303.3997. https://arxiv.org/abs/1303.3997. [Preprint].

[CIT0030] Li H , Handsaker B, Wysoker A, Fennell T, Ruan J, Homer N, Marth G, Abecasis G, Durbin R 1000 Genome Project Data Processing Subgroup. 2009. The sequence alignment/map format and SAMtools. Bioinformatics 25, 2078–2079. 19505943 10.1093/bioinformatics/btp352 PMC2723002

[CIT0031] Li F , Vallabhaneni R, Wurtzel ET. 2008. *PSY3*, a new member of the phytoene synthase gene family conserved in the Poaceae and regulator of abiotic stress-induced root carotenogenesis. Plant Physiology 146, 1333–1345. 18162592 10.1104/pp.107.111120 PMC2259096

[CIT0032] Lin T , Zhu G, Zhang J, et al. 2014. Genomic analyses provide insights into the history of tomato breeding. Nature Genetics 46, 1220–1226. 25305757 10.1038/ng.3117

[CIT0033] Liu J , Van Eck J, Cong B, Tanksley SD. 2002. A new class of regulatory genes underlying the cause of pear-shaped tomato fruit. Proceedings of the National Academy of Sciences, USA 99, 13302–13306. 10.1073/pnas.162485999 PMC130628 12242331

[CIT0034] Luckwill LC. 1943. The genus *Lycopersicon*: an historical, biological, and taxonomic survey of the wild and cultivated tomatoes. Aberdeen: The University Press.

[CIT0035] Mazzucato A , Papa R, Bitocchi E, et al. 2008. Genetic diversity, structure and marker-trait associations in a collection of Italian tomato (*Solanum lycopersicum L.*) landraces. Theoretical and Applied Genetics 116, 657–669. 18193185 10.1007/s00122-007-0699-6

[CIT0036] Mccue GA. 1952. The history of the use of the tomato: an annotated bibliography. Annals of the Missouri Botanical Garden 39, 289–348.

[CIT0037] Menda N , Strickler SR, Edwards JD, et al. 2014. Analysis of wild-species introgressions in tomato inbreds uncovers ancestral origins. BMC Plant Biology 14, 287. 25348801 10.1186/s12870-014-0287-2 PMC4219026

[CIT0038] Mu Q , Huang Z, Chakrabarti M, Illa-Berenguer E, Liu X, Wang Y, Ramos A, van der Knaap E. 2017. Fruit weight is controlled by *Cell Size Regulator* encoding a novel protein that is expressed in maturing tomato fruits. PLoS Genetics 13, e1006930. 28817560 10.1371/journal.pgen.1006930 PMC5560543

[CIT0039] Muggeo VM. 2003. Estimating regression models with unknown break-points. Statistics in Medicine 22, 3055–3071. 12973787 10.1002/sim.1545

[CIT0040] Muños S , Ranc N, Botton E, et al. 2011. Increase in tomato locule number is controlled by two single-nucleotide polymorphisms located near *WUSCHEL*. Plant Physiology 156, 2244–2254. 21673133 10.1104/pp.111.173997 PMC3149950

[CIT0041] Park YH , West MA, St Clair DA. 2004. Evaluation of AFLPs for germplasm fingerprinting and assessment of genetic diversity in cultivars of tomato (*Lycopersicon esculentum L.*). Genome 47, 510–518. 15190368 10.1139/g04-004

[CIT0042] Peakall R , Smouse PE. 2006. GENALEX 6: genetic analysis in Excel. Population genetic software for teaching and research. Molecular Ecology Notes 6, 288–295. 10.1093/bioinformatics/bts460 PMC3463245 22820204

[CIT0043] Peakall R , Smouse PE. 2012. GenAlEx 6.5: genetic analysis in Excel. Population genetic software for teaching and research—an update. Bioinformatics 28, 2537–2539. 22820204 10.1093/bioinformatics/bts460 PMC3463245

[CIT0044] Pons C , Blanca J, Cañizares J, Ziarsolo P, Finckers R, Rambla JL, da Silva G, Zacarías L, Monforte AJ, Granell A. 2017. Variability in fruit ripening within the European traditional pool of tomato varieties. XIV Solanacea and 3rd Cucurbitaceae joint conference SOLCUC2017. Valencia, Spain, 3–6 May 2017, 54.

[CIT0045] Raj A , Stephens M, Pritchard JK. 2014. fastSTRUCTURE: variational inference of population structure in large SNP data sets. Genetics 197, 573–589. 24700103 10.1534/genetics.114.164350 PMC4063916

[CIT0046] Razifard H , Ramos A, Della Valle AL, et al. 2020. Genomic evidence for complex domestication history of the cultivated tomato in Latin America. Molecular Biology and Evolution 37, 1118–1132. 31912142 10.1093/molbev/msz297 PMC7086179

[CIT0047] Rick CM , Fobes JF. 1975. Allozyme variation in the cultivated tomato and closely related species. Bulletin of the Torrey Botanical Club 102, 376–384.

[CIT0048] Rick CM , Zobel RW, Fobes JF. 1974. Four peroxidase loci in red-fruited tomato species: genetics and geographic distribution. Proceedings of the National Academy of Sciences, USA 71, 835–839. 10.1073/pnas.71.3.835 PMC388109 16592148

[CIT0049] Robbins MD , Sim SC, Yang W, Van Deynze A, van der Knaap E, Joobeur T, Francis DM. 2011. Mapping and linkage disequilibrium analysis with a genome-wide collection of SNPs that detect polymorphism in cultivated tomato. Journal of Experimental Botany 62, 1831–1845. 21193580 10.1093/jxb/erq367 PMC3060673

[CIT0050] Rodríguez GR , Muños S, Anderson C, Sim SC, Michel A, Causse M, Gardener BB, Francis D, van der Knaap E. 2011. Distribution of *SUN*, *OVATE*, *LC*, and *FAS* in the tomato germplasm and the relationship to fruit shape diversity. Plant Physiology 156, 275–285. 21441384 10.1104/pp.110.167577 PMC3091046

[CIT0051] Rogers AR , Huff C. 2009. Linkage disequilibrium between loci with unknown phase. Genetics 182, 839–844. 19433632 10.1534/genetics.108.093153 PMC2710162

[CIT0052] Sacco A , Ruggieri V, Parisi M, Festa G, Rigano MM, Picarella ME, Mazzucato A, Barone A. 2015. Exploring a tomato landraces collection for fruit-related traits by the aid of a high-throughput genomic platform. PLoS One 10, e0137139. 26393929 10.1371/journal.pone.0137139 PMC4579088

[CIT0053] Sahagún B. 1577. Historia general de las cosas de Nueva España por el fray Bernardino de Sahagún: el Códice Florentino. Libro X: del pueblo, sus virtudes y vicios, y otras naciones. Florence: Biblioteca Medicea Laurenziana.

[CIT0054] Sanfunentes Echevarrría O. 2006. Europa y su percepción del nuevo mundo a través de las especies comestibles y los espacios americanos en el siglo XVI. Historia 39, 531–556.

[CIT0055] Sato S , Tabata S, Hirakawa H, et al. 2012. The tomato genome sequence provides insights into fleshy fruit evolution. Nature 485, 635–641. 22660326 10.1038/nature11119 PMC3378239

[CIT0056] Seabold S , Perktold J. 2010. Statsmodels: econometric and statistical modeling with Python. In: van der Walt S, Millman J, eds. Proceedings of the 9th Python in Science Conference 2010. Austin, TX, USA, 28 June–3 July 2010, 57–61.

[CIT0057] Sim SC , Van Deynze A, Stoffel K, et al. 2012 *a*. High-density SNP genotyping of tomato (*Solanum lycopersicum* L.) reveals patterns of genetic variation due to breeding. PLoS One 7, e45520. 23029069 10.1371/journal.pone.0045520 PMC3447764

[CIT0058] Sim SC , Durstewitz G, Plieske J, et al. 2012 *b*. Development of a large SNP genotyping array and generation of high-density genetic maps in tomato. PLoS One 7, e40563. 22802968 10.1371/journal.pone.0040563 PMC3393668

[CIT0059] Sim SC , Robbins MD, Van Deynze A, Michel AP, Francis DM. 2011. Population structure and genetic differentiation associated with breeding history and selection in tomato (*Solanum lycopersicum* L.). Heredity 106, 927–935. 21081965 10.1038/hdy.2010.139 PMC3186243

[CIT0060] Soyk S , Lemmon ZH, Oved M, et al. 2017. Bypassing negative epistasis on yield in tomato imposed by a domestication gene. Cell 169, 1142–1155.e12. 28528644 10.1016/j.cell.2017.04.032

[CIT0061] Soyk S , Lemmon ZH, Sedlazeck FJ, Jiménez-Gómez JM, Alonge M, Hutton SF, Van Eck J, Schatz MC, Lippman ZB. 2019. Duplication of a domestication locus neutralized a cryptic variant that caused a breeding barrier in tomato. Nature Plants 5, 471–479. 31061537 10.1038/s41477-019-0422-z

[CIT0062] Tanksley SD , McCouch SR. 1997. Seed banks and molecular maps: unlocking genetic potential from the wild. Science 277, 1063–1066. 9262467 10.1126/science.277.5329.1063

[CIT0063] Virtanen P , Gommers R, Oliphant TE, et al. 2020. SciPy 1.0: fundamental algorithms for scientific computing in Python. Nature Methods 17, 261–272. 32015543 10.1038/s41592-019-0686-2 PMC7056644

[CIT0064] Waskom M. 2021. Seaborn: statistical data visualization. Journal of Open Source Software 6, 3021.

[CIT0065] Williams CE , Clair DA. 1993. Phenetic relationships and levels of variability detected by restriction fragment length polymorphism and random amplified polymorphic DNA analysis of cultivated and wild accessions of *Lycopersicon esculentum*. Genome 36, 619–630. 18470012 10.1139/g93-083

[CIT0066] Xiao H , Jiang N, Schaffner E, Stockinger EJ, van der Knaap E. 2008. A retrotransposon-mediated gene duplication underlies morphological variation of tomato fruit. Science 319, 1527–1530. 18339939 10.1126/science.1153040

[CIT0067] Xu C , Liberatore KL, MacAlister CA, et al. 2015. A cascade of arabinosyltransferases controls shoot meristem size in tomato. Nature Genetics 47, 784–792. 26005869 10.1038/ng.3309

[CIT0068] Yuste-Lisbona FJ , Fernández-Lozano A, Pineda B, et al. 2020. ENO regulates tomato fruit size through the floral meristem development network. Proceedings of the National Academy of Sciences, USA 117, 8187–8195. 10.1073/pnas.1913688117 PMC7148573 32179669

[CIT0069] Zheng X , Levine D, Shen J, Gogarten SM, Laurie C, Weir BS. 2012. A high-performance computing toolset for relatedness and principal component analysis of SNP data. Bioinformatics 28, 3326–3328. 23060615 10.1093/bioinformatics/bts606 PMC3519454

