# Correction to: European traditional tomatoes galore: a result of farmers’ selection of a few diversity-rich loci

**DOI:** 10.1093/jxb/erac313

**Published:** 2022-08-08

Jose Blanca, Clara Pons, Javier Montero-Pau, David Sanchez-Matarredona, Peio Ziarsolo, Lilian Fontanet, Josef Fisher, Mariola Plazas, Joan Casals, Jose Luis Rambla, Alessandro Riccini, Samuela Palombieri, Alessandra Ruggiero, Maria Sulli, Stephania Grillo, Angelos Kanellis, Giovanni Giuliano, Richard Finkers, Maria Cammareri, Silvana Grandillo, Andrea Mazzucato, Mathilde Causse, Maria José Díez, Jaime Prohens, Dani Zamir, Joaquin Cañizares, Antonio Jose Monforte, Antonio Granell, European traditional tomatoes galore: a result of farmers’ selection of a few diversity-rich loci, Journal of Experimental Botany, Volume 73, Issue 11, 2 June 2022, Pages 3431–3445, https://doi.org/10.1093/jxb/erac072.

In the originally published version of this manuscript, the surname of author Samuela Palombieri was incorrectly given as Pombarella.

This error has been corrected.

